# Enhancing E-cadherin expression via promoter-targeted miR-373 suppresses bladder cancer cells growth and metastasis

**DOI:** 10.18632/oncotarget.21400

**Published:** 2017-09-30

**Authors:** Qingsong Zhang, Chenghe Wang, Shuo Miao, Chuanchang Li, Zhong Chen, Fan Li

**Affiliations:** ^1^ Department of Urology, Tongji Hospital, Tongji Medical College, Huazhong University of Science and Technology, Wuhan 430030, Hubei, China; ^2^ Department of Urology, Ruijin Hospital, School of Medicine, Shanghai Jiaotong University, Shanghai 200025, China; ^3^ Department of Pharmacology, Tongji Medical College, Huazhong University of Science and Technology, Wuhan 430030, Hubei, China

**Keywords:** RNA activation, MiR-373, bladder cancer, proliferation, metastasis

## Abstract

Previous studies showed that miR-373 had the capacity to induce tumor suppressor gene E-cadherin expression in prostate cancer cells. However, whether miR-373 can activate the expression of E-cadherin in human bladder cancer (BCa) cells and inhibit cells remains to be elucidated. Here, we found that both miR-373 and E-cadherin were low expressed in BCa tissues and cell lines, and significantly correlated with tumor stage, grade, and lymph node metastasis. In addition, decreased E-cadherin expression or low expression of both miR-373 and E-cadherin is associated with poor overall survival in patients with BCa. Transfection of miR-373 into BCa cells readily activated E-cadherin expression by targeting promoter. Moreover, miR-373 exhibited robust capacity to inhibit cells proliferation, suppress migration and invasion by enhancing E-cadherin expression, and significantly suppress the growth of xenografts and metastasis in nude mice. Altogether, our findings indicate that miR-373 may as a tumor suppressor in BCa by activating E-cadherin expression.

## INTRODUCTION

In the urinary system, bladder cancer (BCa) is the most common malignant tumor. There are approximate 60,490 male and 18,540 female newly diagnosed cases and 16,870 deaths both male and female in United States in 2017 [1]. Furthermore, BCa is the primary cause of death among urinary tumors in China [2]. Although more than 70% of BCa patients are non-muscular invasive cancer based on initial diagnosis, they have a high risk of recurrence and progression after local therapies [3]. The newly diagnosed muscle invasive cases are usually managed by radical surgery, radiotherapy and even systemic treatment, but the prognosis is still poor [4, 5]. Down-regulation expression of specific tumor suppressor genes was confirmed to largely contribute to BCa initiation, proliferation and metastasis, so targeted gene therapy would be an effective strategy for its treatment [6].

Epithelial cadherin (E-cadherin) belongs to cadherin family and locates on 16q23 [7]. As an essential tumor suppressor gene, E-cadherin is one of the most important genes in maintaining epithelial cell-cell adhesion and normal tissue structure [8]. Loss or decrease of E-cadherin could promote epithelial-mesenchymal transition (EMT) and induce cancer cells invasion and migration [9]. Moreover, E-cadherin plays a key role in tumor progression, survivin expression, tumor size and overall survival in human BCa [10].

MicroRNAs (miRNAs) are a cluster of endogenous small non-coding RNAs (ncRNAs) and related to cell proliferation, development and apoptosis [11]. RNA activation (RNAa) is a newly detected mechanism for the positive regulation gene expression at the transcriptional level, in which endogenous miRNAs or synthetic ordouble-stranded RNAs (dsRNAs) are complementary to specific promoter sequences of target genes [12, 13]. There is increasing evidences showed that some of the specific tumor suppressor genes activated by RNAa can cause anti-tumor effects in a variety of cancer cells [14–17]. It was manifested that a putative miR-373 could induce E-cadherin expression by targeting specific promoter sequences in prostate cancer PC-3 cells [13]. Moreover, miR-373 was demonstrated to be low expressed in a variety of cancers and restoration of it could significantly inhibit tumor progression [18–21].

In the present study, we examined the role of miR-373 in BCa cells development. We found that miR-373 could robustly enhance E-cadherin expression in BCa cells by targeting its promoter, and inhibit cells growth and metastasis. Our results indicate that miR-373 may serve as a new candidate anti-cancer drug for BCa management.

## RESULTS

### MiR-373 and E-cadherin expressions are reduced in BCa tissues and cells and are correlated with BCa progression

**Figure 1:**
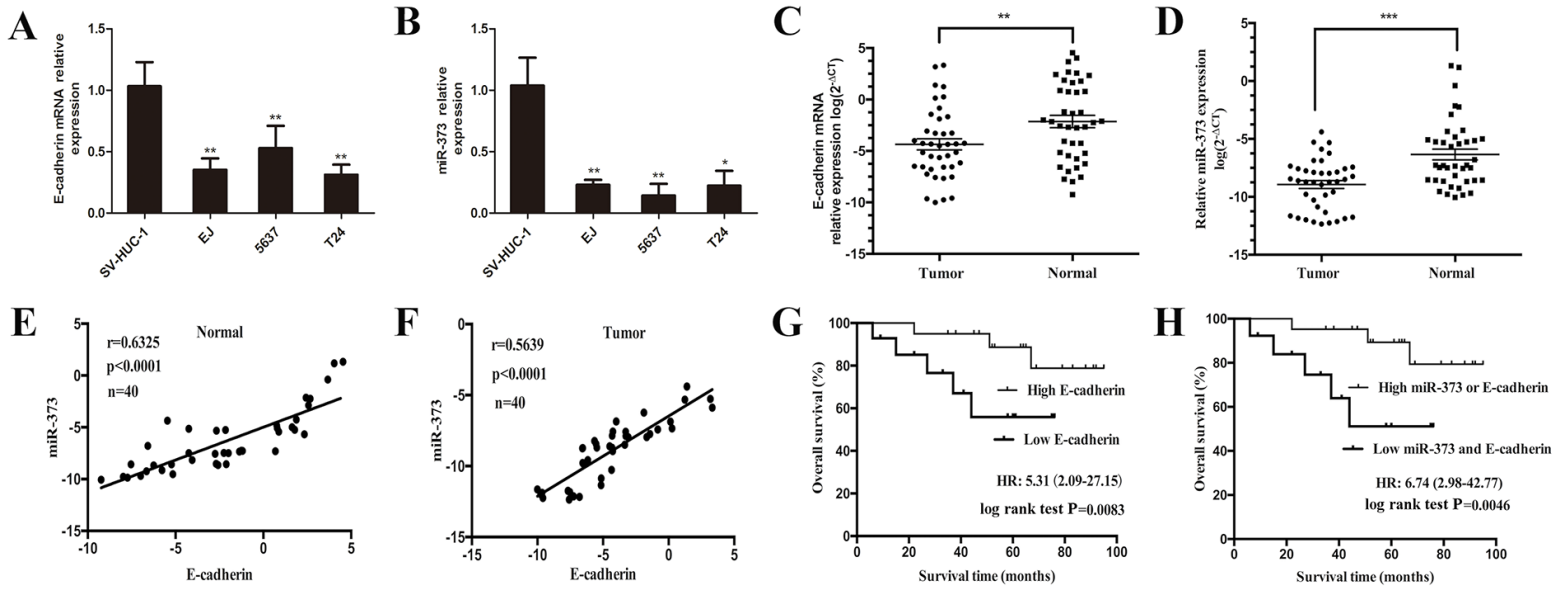
Combined low expression levels of miR-373 and E-cadherin were associated with poor overall survival in patients with BCa **(A)** E-cadherin and **(B)** miR-373 expressions were reduced in EJ, 5637 and T24 cell lines compared with primary normal human bladder epithelial cells (SV-HUC-1), (*P<0.05, **P<0.01). Both **(C)** E-cadherin and **(D)** miR-373 expressions were reduced in human BCa tissues compared with the expressions in adjacent normal tissues. Both **(E)** and **(F)** showed positive correlation between miR-373 and E-cadherin expression levels in BCa and adjacent normal tissues. Statistical analysis was performed using the Pearson correlation coefficient analysis, with r and P values as indicated. **(G)** Kaplan-Meier curves and log-rank test showed that high E-cadherin expression was associated with favorable overall survival when compared with low E-cadherin expression (P=0.0083). **(H)** Patients with low expression levels of miR-373 and E-cadherin survived significantly shorter than those with high miR-373 or E-cadherin expression levels did (P=0.0046).

To assess the potential value of miR-373 and E-cadherin in BCa, we detected both miR-373 and E-cadherin expression in BCa cells and clinical tissues. The expression levels of miR-373 and E-cadherin were decreased in 3 BCa cell lines including EJ, 5637 and T24 cells compared with SV-HUC-1 cells (Figure 1A, 1B). Then, we evaluated the miR-373 and E-cadherin expression levels in 40 pairs of BCa and the corresponding adjacent normal tissues. The outcomes showed that miR-373 and E-cadherin expressions were also significantly lower in tumor tissues than adjacent normal tissues (Figure 1C, 1D). Furthermore, we analyzed the relationships between miR-373, E-cadherin expression and the clinicopathologic factors of BCa, as shown in Table 1, miR-373 and E-cadherin expressions (T/N) were related to tumor stage, grade, and lymph node metastasis (P < 0.05). These suggest that miR-373 and E-cadherin may act as tumor suppressors in BCa.

**Table 1:** Relationship between expression of miR-373, E-cadherin, and clinicopathologic factors in patients with BC (n=40)

Parameter	Number of case	miR-373 expression	*P* value	E-cadherin expression	*P* value
Age, y					
>65	24	0.54 ± 0.21	0.78	0.66 ± 0.11	0.87
<65	16	0.58 ± 0.23		0.63 ± 0.13	
Sex					
Male	27	0.49 ± 0.25	0.53	0.68 ± 0.17	0.91
Female	13	0.52 ± 0.22		0.70 ± 0.24	
Tumor diameter, cm					
<3	22	0.62 ± 0.24	0.093	0.68 ± 0.15	0.11
>3	18	0.57 ± 0.27		0.59 ± 0.23	
Category					
Ta	23	0.67 ± 0.26	0.036	0.77 ± 0.25	0.014
T1	11	0.54 ± 0.17		0.60 ± 0.16	
T2-4	6	0.44 ± 0.11		0.48 ± 0.12	
Grade					
G1	28	0.56 ± 0.15	0.018	0.65 ± 0.17	0.032
G2/G3	12	0.33 ± 0.12		0.44 ± 0.26	
Node metastases					
No	36	0.58 ± 0.20	0.02	0.73 ± 0.14	0.028
Yes	4	0.34 ± 0.13		0.53 ± 0.17	

### MiR-373 expression is positively associated with E-cadherin expression

Both miR-373 and E-cadherin were verified to be relate to BCa progression, the Pearson correlation coefficient analysis was applied to detect the relationship between miR-373 and E-cadherin expression levels. Result showed that the expression level of miR-373 was positively correlated with the level of E-cadherin expression in BCa and adjacent normal tissues (Figure 1E; r = 0.63, P<0.0001 and Figure 1F; r = 0.56, P<0.0001).

### Low level of E-cadherin expression or decreased expression level of both miR-373 and E-cadherin is correlated with poor overall survival (OS) in patients with BCa

First, we analyzed the relationship between E-cadherin expression and OS of BCa patients. Choosing the median-fold change (T/N) in E-cadherin expression as the cutoff value, the OS of patients with high E-cadherin expression was significantly longer than those of low E-cadherin expression (Figure 1G; HR = 5.31, 95% CI: 2.09–27.15; P = 0.0083) [22]. Then a multivariate Cox analysis was performed to assess whether E-cadherin is an independent prognostic factor for BCa. The report showed that low E-cadherin expression in BCa was related to poor prognosis for OS (HR = 3.98, 95% CI: 1.21–17.02; P = 0.021), independent of other cliniclopathologic variables (Table 2), which suggesting that E-cadherin might be served as an independent prognostic factor for BCa. We next analyzed selected patients’ subgroups based on the combination of miR-373 and E-cadherin expression. Based on the median-fold change in miR-373 and E-cadherin expression, BCa patients were divided into two subgroups (high miR-373 or E-cadherin group, low miR-373 and E-cadherin group). According to the levels of miR-373 and E-cadherin expression, Kaplan-Meier curves suggested that BCa patients with high levels miR-373 or E-cadherin expression survived longer than those with low levels of miR-373 and E-cadherin expression did (Figure 1H; HR = 6.74, 95% CI: 2.98–42.77; P = 0.0046). Similarly, the multivariate Cox regression model indicated that low expression level of miR-373 and E-cadherin in BCa was correlated with a poor prognosis for OS (HR = 5.29, 95% CI: 2.02–33.96; P = 0.014), independent of other clinicopathologic factors (Table 2).

**Table 2:** Univariate and multivariate analysis of various prognostic variables and overall survival in patients with BC (n=40)

Variables (and stratification)	Univariate analysis		Multivariate analysis	
	HR(95% CI)	*P* value	HR(95% CI)	*P* value
Age (>65 vs. <65 y)	1.26(0.32-3.74)	0.63	/	/
Sex (male vs. female)	1.98(0.57-3.14)	0.44	/	/
BMI (>25.51 vs. <25.51 kg/m^2^)	2.32(0.97-6.22)	0.097	/	/
Stage (T2–4 vs. Ta-1)	6.14(1.55-23.69)	0.023	4.53(1.17-14.22)	0.052
Grade (G2-3 vs. G1)	6.38(1.37-27.25)	0.0092	5.16(1.32-16.79)	0.037
Tumor size (>3 vs. <3 cm)	3.75(2.23-19.49)	0.064	/	/
Node metastases (yes vs. no)	6.63(1.17-37.05)	0.011	5.37(1.08-21.10)	0.041
miR-373 expression (low vs. high)	2.27(1.43-4.77)	0.17	/	/
E-cadherin expression (low vs. high)	5.31(2.09-27.15)	0.0083	3.98(1.21-17.02)	0.021
Low miR-373 and E-cadherin vs. high miR-373 or E-cadherin	6.74(2.98-42.77)	0.0046	5.29(2.02-33.96)	0.014

### MiR-373 activates E-cadherin expression in BCa cells

**Figure 2:**
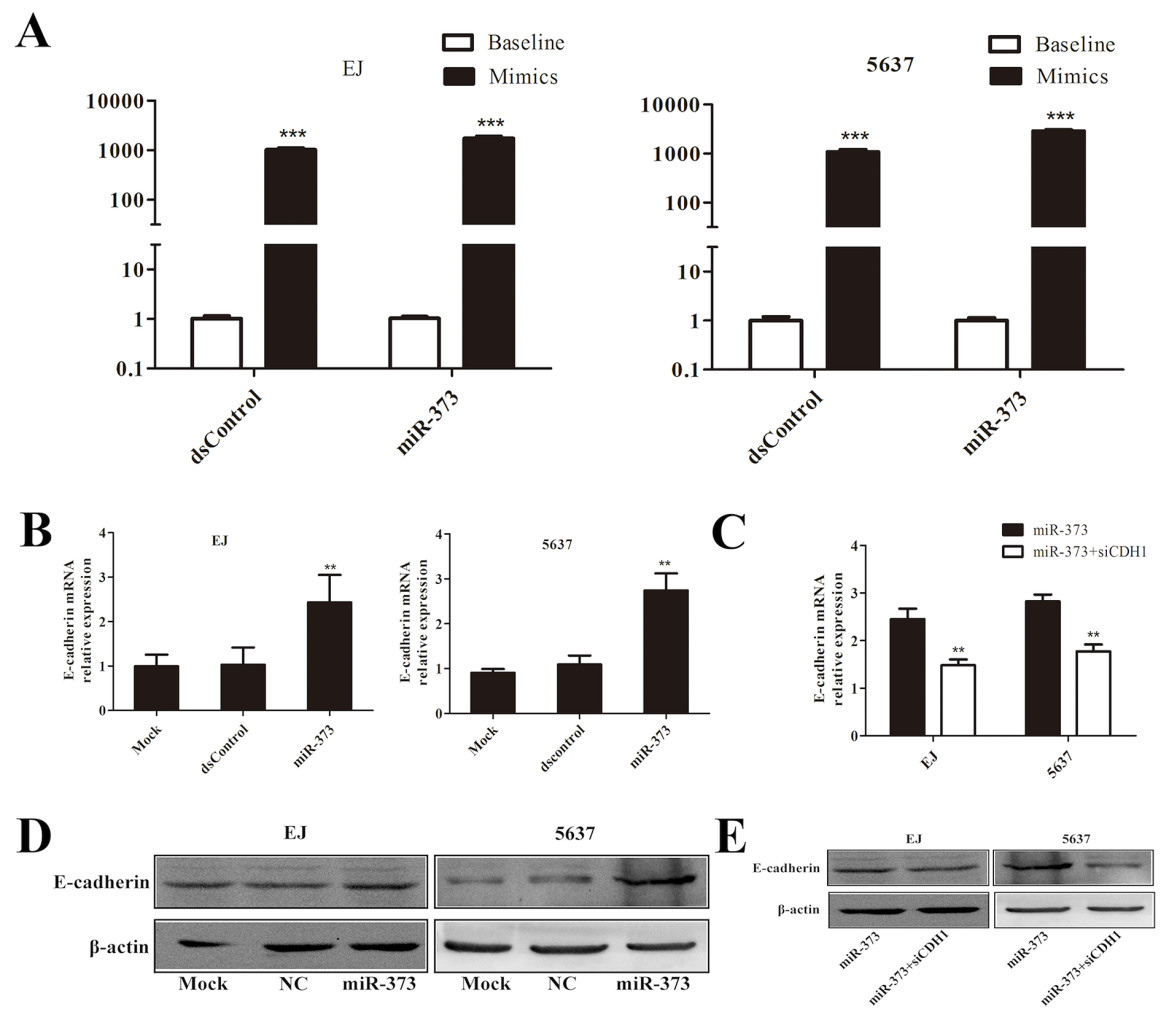
MiR-373 can effectively activate E-cadherin expression both in EJ and 5637 cells **(A)** Transfection ratio was tested by qRT-PCR, dsControl and miR-373 were significantly increased in mimic group than baseline in EJ and 5637 cells. **(B)** Induction of E-cadherin mRNA expression was detected by qRT-PCR in both EJ and 5637 cells. The results were normalized to GAPDH. **P<0.01 compared with mock and dsControl groups. **(D)** Induction of E-cadherin protein expression was detected by Western blot analysis. β-actin levels were also detected and served as an internal control. **(C and E)** Expression of E-cadherin mRNA and protein was detected by qRT-PCR and Western blot respectively. GAPDH and β-actin served as internal control respectively. E-cadherin expression was dramatically decreased in co-transfection group compared with the miR-373 group.

To observe the effects of miR-373 on E-cadherin in BCa cells, we transfected miR-373 mimic into BCa cells and detected E-cadherin expression 48-72 h later. The cells transfection ratios were tested by quantitative Real-Time PCR (qRT-PCR) (Figure 2A). The expression of E-cadherin mRNA was significantly increased after miR-373 mimic transfection (Figure 2B). Induction of E-cadherin protein expression was further confirmed by Western blot (Figure 2D). Moreover, we co-transfected siCDH1 (silencing E-cadherin expression) and miR-373 mimic into BCa cells and found that E-cadherin expression was dramatically abrogated compared with miR-373 group (Figure 2C, 2E).

### MiR-373 interacts with E-cadherin gene promoter

**Figure 3:**
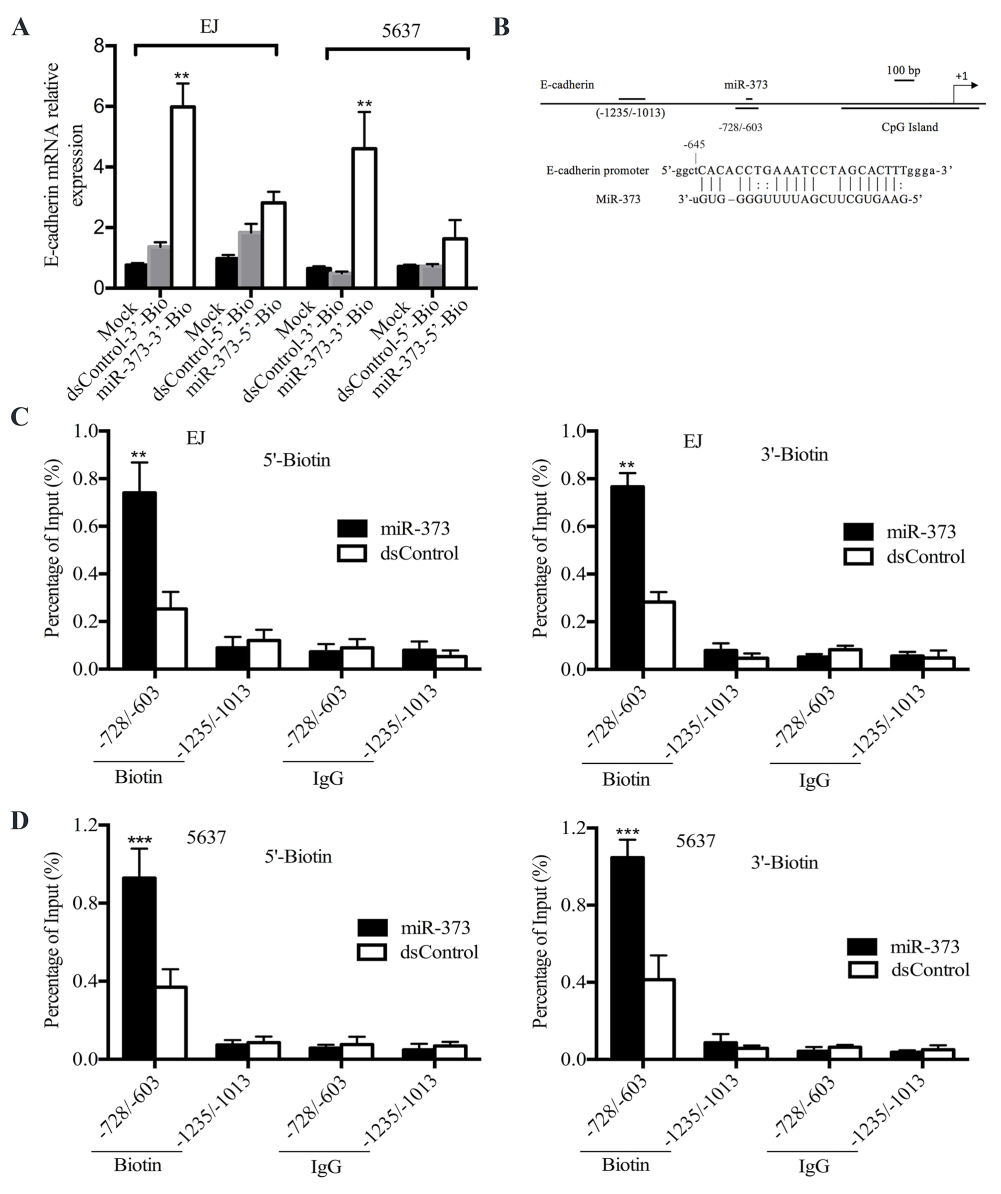
miR-373 interacts with E-cadherin promoter **(A)** MiR-373 and dsControl covalently linked to biotin at the 3′-end or at the 5′-end of the strand. And linking a biotin group to the 5′-end of the strand of miR-373 abolished activation of E-cadherin, whereas biotin labeling at the 3′-end did not significantly affect miR-373 activity (**P<0.01). **(B)** Schematic illustration of the primers capable of amplifying E-cadherin promoter at different regions. Locations are shown relative to the TSS. **(C and D)** The biotin-labeled miR-373 pulled down promoter proximal DNA (-728/-603) more effectively than the dsControl RNA did (**P<0.01, ***P<0.001). In contrast, there was no difference in the binding of miR-373 and dsControl RNAs to the DNA upstream of the E-cadherin promoter that served as a negative control.

Others and we have found that the sequences of miRNAs or dsRNAs are complementary to target genes specific promoter sequences is one of the possible mechanisms RNAa [23–25]. To identify whether a putative miR-373 activating E-cadherin in BCa cells by targeting specific site in the promoter, we conducted ChIP assay by using biotinylated miR-373 which was transfected into EJ and 5637 cells. The biotin was covalently linked to either the 5′-end or the 3′-end of miR-373 and dsControl antisense. Before they were used in ChIP assay, we first tested RNAa activity of these biotinylated miR-373 in EJ and 5637 cells. As shown in Figure 3A, linking a biotin group to the 5′-end of miR-373 (miR-373-5′-Bio) abolished activation of E-cadherin, whereas 3′-end of miR-373 (miR-373-3′-Bio) did not affect its activating activity.

After 72h later, miR-373 or dsControl was transfected alone, the specific DNA was pulled down through with a well-characterized biotin antibody and then amplified by qRT-PCR with specific primers at different locations (Figure 3B). One set of primers amplifying the E-cadherin promoter from -1235bp to -1013bp relative to the transcription start site (TSS) acted as a negative control. As shown in Figure 3C and 3D, both 3′-end and 5′-end of the biotin-labelled miR-373 pulled down promoter proximal DNA (from -728 to -603) more effectively than the dsControl did. On the contrary, there was no significant difference in the binding of miR-373 and dsControl RNAs to the negative control (-1235 to -1013). These outcomes suggest that miR-373 induces gene expression by directly interacting with the E-cadherin promoter.

### MiR-373 inhibits BCa cells proliferation mainly through activating E-cadherin expression

**Figure 4:**
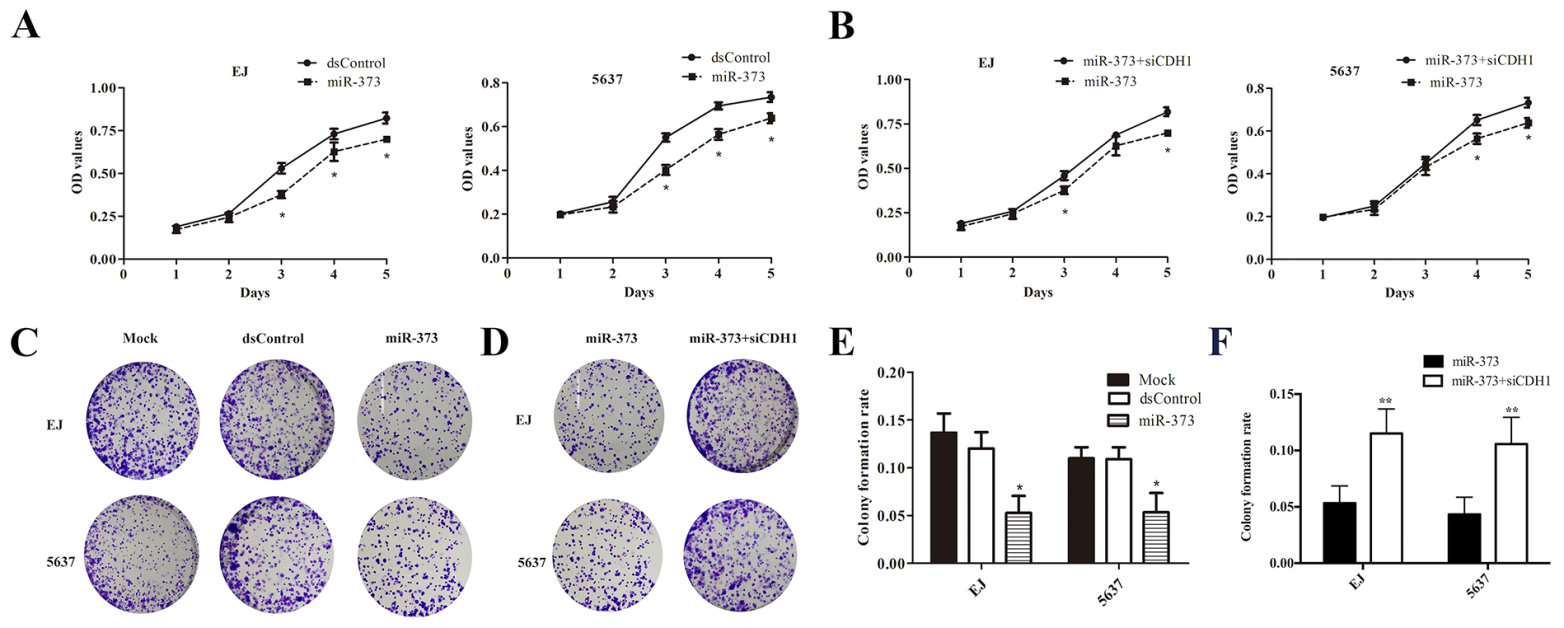
MiR-373 inhibited cells proliferation in both EJ and 5637 cells **(A)** Viable cells were measured from day 1 to 5 following miR-373 transfection using MTS. Results were plotted as OD values. *P<0.05 reveals the corresponding group compared with dsControl at the same time point. **(B)** After co-transfection with siCDH1, cell proliferation increased gradually *P<0.05. **(C and D)** Representative photographs of the colony formation assay. **(E)** Quantification of the cell colonies formation. *P<0.05 compared with dsControl group. **(F)** Similarly, quantification of the cell colonies formation. While co-treatment with siCDH1, the amounts of colony were much more compared with miR-373 groups (**P<0.01).

In order to elucidate the inhibitory effect of miR-373 on BCa cells, we enforced miR-373 expression in EJ and 5637 cells via miRNA mimics. Both EJ and 5637 cells displayed progressive retarded growth compared to dsControl group from 48h after transfection of miR-373 mimics measured by MTS assay (Figure 4A). And knockout of E-cadherin evidently weakened the anti-proliferative effect regulated by miR-373 in both cell lines (Figure 4B). Furthermore, we performed the colony formation assay and detected that miR-373 transfected cells formed colonies significantly fewer and smaller than dsControl group (Figure 4C). In addition, the colony formation rates of miR-373 transfected both EJ and 5637 cells were significantly lower than dsControl treatment (Figure 4E). And the colony formation ability of the BCa cells was restored after co-transfection of siCDH1 compared with miR-373 group (Figure 4D and 4F).

**Figure 5:**
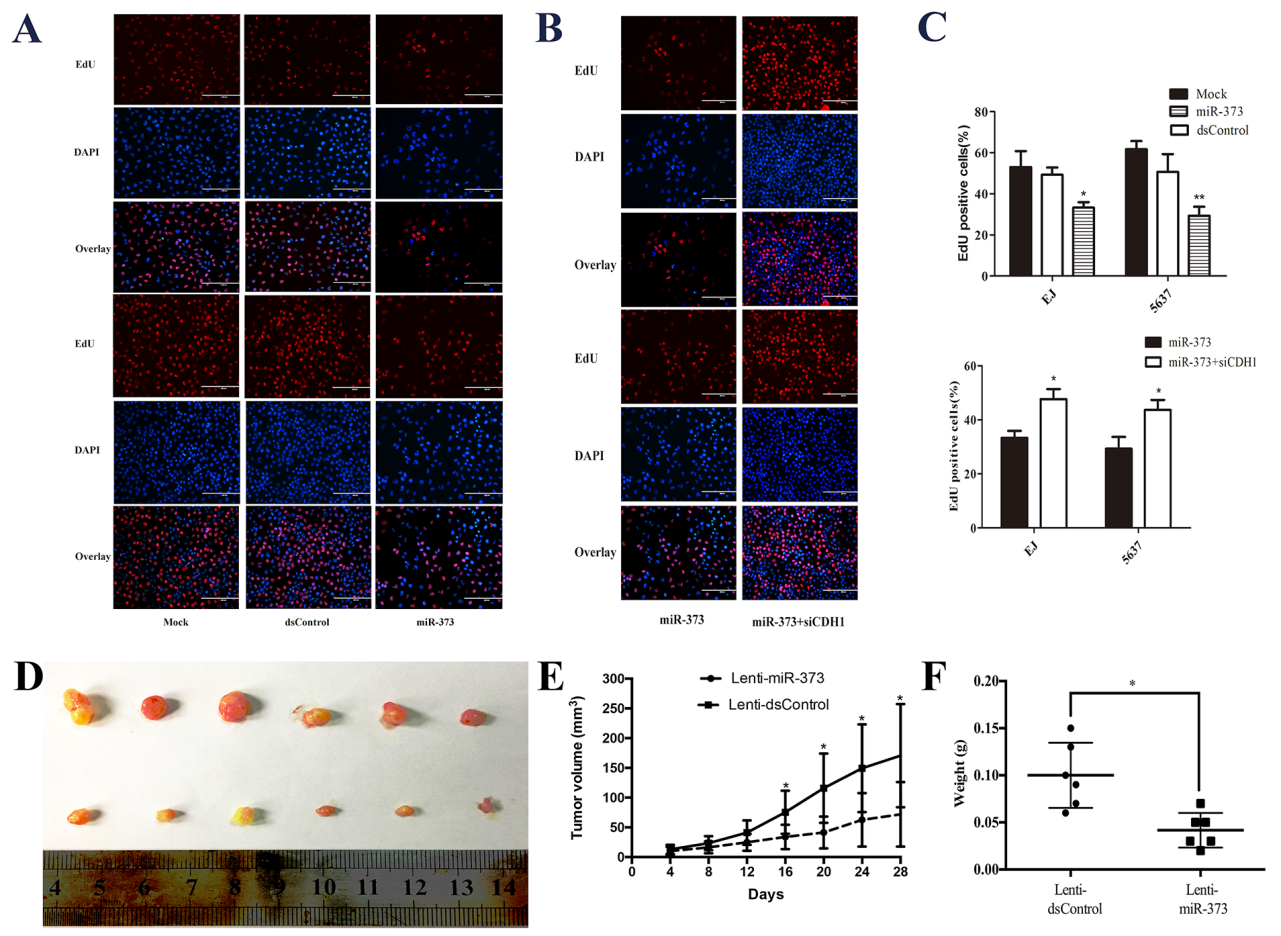
MiR-373 inhibited BCa cells proliferation *in vitro* and *in vivo* **(A and B)** Representative micrographs of EdU-positive cells (red). The nucleus was stained with DAPI (blue). Overexpression of miR-373 inhibited cell proliferation in both EJ and 537 cells. While co-treatment with siCDH1, the cell proliferation was restored. **(C)** Quantification of EdU-positive cells (*P<0.05, **P<0.01). **(D)** Photographs of tumors excised 28 days after inoculation of stably transfected EJ cells into nude mice. **(E)** Mean tumor volume measured by caliper on the indicated days. *P<0.05 compared with Lenti-dsControl group. **(F)** Tumor weight of each nude mouse at the end of 28 days. *P<0.05 compared to Lenti-dsControl group.

To further confirm the changes of cell proliferation induced by miR-373, an EdU assay was conducted and the outcomes showed over-expression of miR-373 suppressed cells proliferation in two BCa cell lines (Figure 5A and 5C). While co-treatment with siCDH1, the cells proliferation ability was restored (Figure 5B and 5C). Then, the EJ cells were stably transfected miR-373 or dsControl, and the transfected EJ cells were used to generate the xenograft model in nude mice. As shown in Figure 5D, Lenti-miR-373 significantly inhibited xenograft tumor growth. Besides, the average tumor volume and weight in Lenti-miR-373 group was remarkably decreased and lightened compared with the Lenti-dsControl group at 4 weeks post injection, respectively (Figure 5E and 5F).

### MiR-373 inhibits downstream genes’ expression of E-cadherin

**Figure 6:**
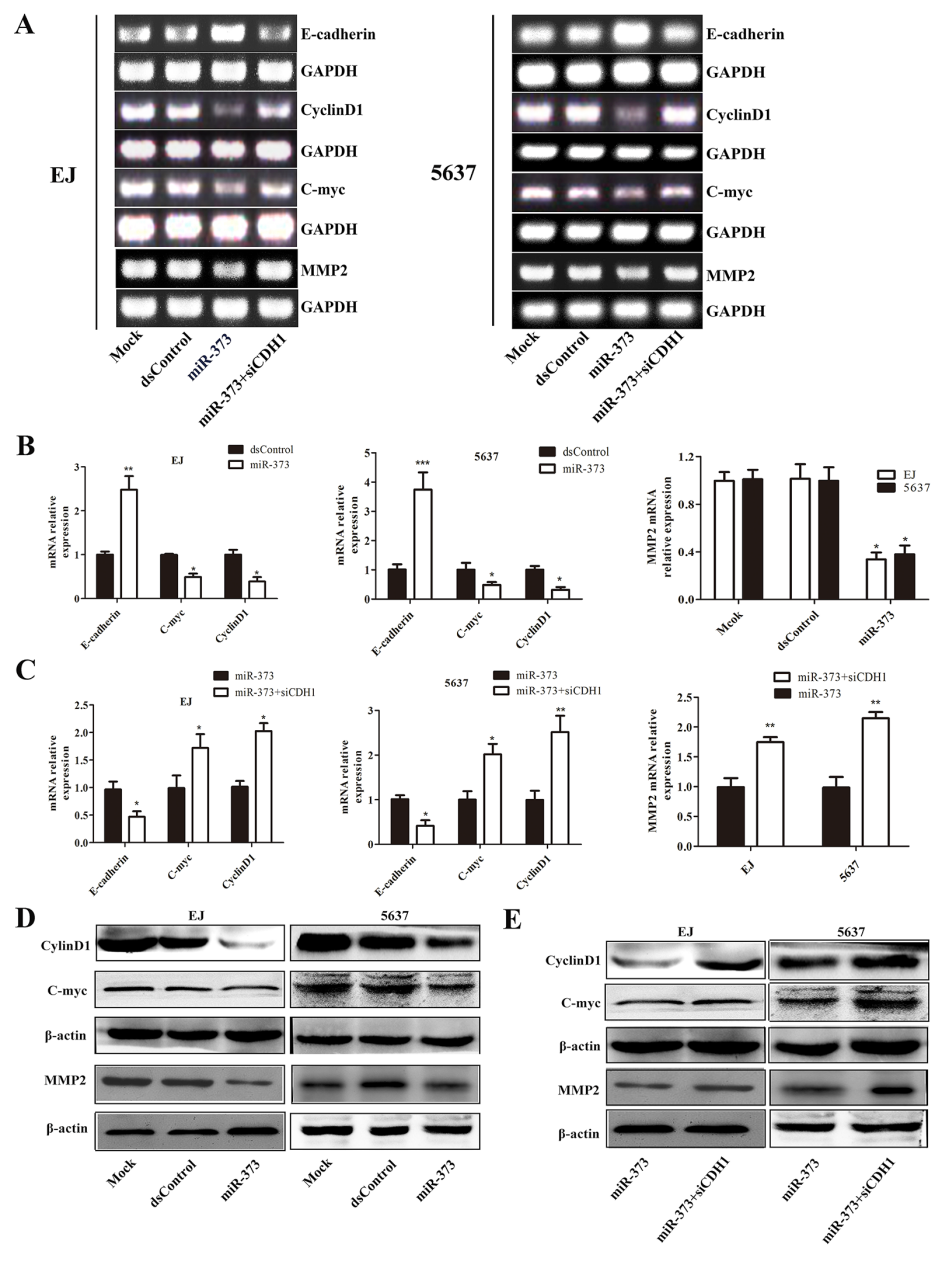
MiR-373 inhibits E-cadherin’s downstream genes Cyclin D1, C-myc and MMP2 expression The expression of E-cadherin, Cyclin D1, C-myc and MMP2 mRNAs were detected by reverse transcription PCR (RT-PCR) **(A)** and qRT-PCR **(B and C)**. GAPDH served as a loading control. MiR-373 increased E-cadherin and suppressed CyclinD1, C-myc and MMP2 mRNAs expression. After co-transfected with siCDH1, miR-373 failed to up-regulate E-cadherin or down-regulated Cyclin D1, C-myc and MMP2 mRNA levels (*P<0.05, **P<0.01 ***P<0.001). **(D)** Expression of Cyclin D1, C-myc and MMP2 protein were detected by Western blot. β-actin served as a loading control. Cyclin D1, C-myc and MMP2 proteins were lower compared with mock and dsControl group in EJ and 5637 cells. **(E)** Co-transfected with siCDH1 contributed to the targeted genes significant increase in two cell lines.

We further detected the influences of miR-373 on the expression of genes associated with cell proliferation and migration in two BCa cell lines. The results showed miR-373 caused a marked decrease of CyclinD1, C-myc and MMP2 mRNA levels in both EJ and 5637 cells (Figure 6A and 6B). The protein levels of these genes were further identified by western blotting and the variation trend was highly consistent with the trend of mRNA levels (Figure 6D). Then, we blocked E-cadherin expression and examined the expression of associated genes. As shown in Figure 6A and 6C, miR-373 failed to decrease Cyclin D1, C-myc and MMP2 mRNA levels after siCDH1 co-transfection. The protein analysis of immunoblot further proved that (Figure 6E).

### MiR-373 suppresses BCa cells migration and invasion primarily via activating E-cadherin expression

**Figure 7:**
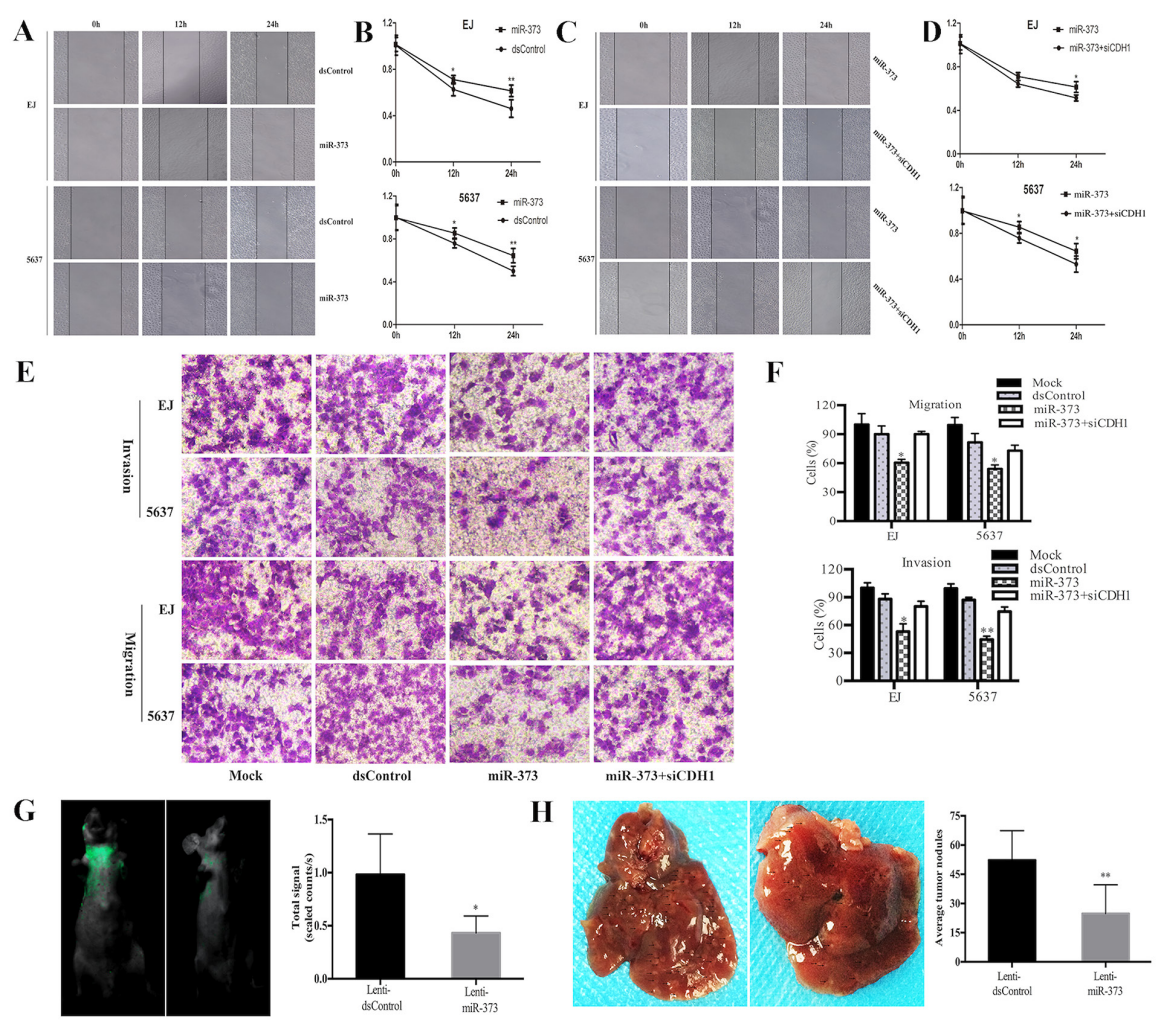
MiR-373 suppresses BCa cells migration and invasion EJ and 5637 cells were transfected with 50 nM of miR-373 and dsControl for 72 h. **(A and C)** Representative wound healing images were pictured at 0, 12 and 24 h. **(B and D)** The relative distances between wound edges of BCa cells at 0, 12 and 24 h. MiR-373 led to retarded wound closing compared with dsControl group from 12h in both EJ and 5637 cells. While siCDH1 co-transfected EJ and 5637 cells recovered to close the wound faster within 24h compared to miR-373 treatment alone (*P<0.05, **P<0.01). **(E)** Representative photographs of transwell assay (×200). **(F)** Number of migrated and invaded cells was quantified. Results are plotted as percent (%) relative to dsControl group. MiR-373 exerted a potent inhibiting effect on migration and invasion of the both cell lines (*P<0.05, **P<0.01). And depletion of E-cadherin dramatically restored cells invasion and migration capacity (*P<0.05). **(G)** Representative bioluminescent images of lungs of nude mice at the 30th days after intravenous injection. Quantification analysis of fluorescence signal from captured bioluminescence images. *P<0.05 compared to Lenti-dsControl group. **(H)** Representative photographs of metastatic nodules in the liver. Number of nodules was quantified. **P<0.01 compared to Lenti-dsControl group.

As we all know, E-cadherin could inhibit tumor cells’ EMT. Hence, we tested the effects of miR-373 in migration and invasion capacities of BCa cells. Wound healing assay indicated that transfection of miR-373 led to slower wound closing speed than dsControl group from 12h in tested cells (Figure 7A and 7B). Furthermore, compared to individual transfect miR-373, siCDH1 co-transfected EJ and 5637 cells recovered to close the wound fast within 24 h (Figure 7C and 7D). Next, the transwell assay was performed to further evaluate cells migration and invasion capability. Compared with dsControl, miR-373 displayed a potent inhibition on migration of the tested cell lines. Likewise, invasion capability of BCa cells was tested by Matrigel invasion chamber assay. As speculation, miR-373 can markedly attenuate EJ and 5637 cells’ invasion ability compared with dsControl group. Moreover, depletion of E-cadherin dramatically restored cells invasion and migration compared with miR-373 transfection alone (Figure 7E and 7F). These results demonstrated that miR-373 could attenuate the migration and invasion ability of BCa cells mainly by inducing E-cadherin expression.

Bioluminescence imaging showed that fluorescence signal in Lenti-miR-373 group was remarkably weaker than Lenti-dsControl group, which means that less metastasis was occurred in lung tissue after miR-373 up-regulation (Figure 7G). Otherwise, we also noticed that the number of metastatic nodules in the liver was significantly reduced in Lenti-miR-373 groups compared with Lenti-dsControl group (Figure 7H).

## DISCUSSION

In the present study, we found that the miR-373 activated E-cadherin expression via directly targeting the promoter to study its inhibition on the cell proliferation, invasion and migration in BCa. Besides, miR-373 could also dramatically inhibit the growth of BCa xenografts and metastasis in nude mice. And several proliferation related genes (Cyclin D1 and C-myc) were decreased following miR-373 transfection. The metastasis associated gene MMP2 was also decreased after miR-373 treatment. Most importantly, after co-transfection with siCDH1, the anti-tumor effects of miR-373 were markedly weakened, which indicated that miR-373 inhibited BCa cells proliferation and metastasis *in vitro* and *in vivo* mainly by activating E-cadherin expression.

As far as know, this is the first study of the role of miR-373 role in BCa. MiR-373 act as a tumor suppressor role during BCa progression by recovering E-cadherin expression which leads to down-regulation of Cyclin D1, C-myc and MMP2, therefore inhibits cells proliferation, migratory and invasive capacity. Although the clinicopathological role of miR-373 has not been estimated in BCa, down-regulation of miR-373 has been discovered in several different types of cancers [18–21, 26]. However, opposite results are also found for miR-373 in several cancer types [22, 27, 28]. Our results also indicated that knockout of E-cadherin also induces stronger proliferation ability and higher cell motility in EJ and 5637 cells. Briefly, E-cadherin plays an important role in the miR-373 suppressed EMT signaling pathway [29]. Conversely, E-cadherin deletion in BCa specimens have been associated with the tumor recurrence, metastasis and poor survival of BCa patients [30–32].

RNAa is a gene transcriptional activation phenomenon discovered in recent years, which leads to changes in chromatin structure through targeting gene promoter-specific sequences induced by small ncRNAs [12, 33]. RNAa requires complementary sequences between dsRNA or miRNA and its targeted genes, which may be one of the most important possible mechanisms. Huang et al. [23] have proved that 3 endogenous miRNAs can enrich RNA polymerase II (RNAP II) and trimethylation of histone 3 at lysine 4 (H3K4me3) at the transcription start site through direct interaction with the promoter region of Ccnb1. Functionally, miRNA activated Ccnb1 expression and regulated tumor progression. And we also proved that miR-1236, miR-1180 and miR-370 could activate the expression of suppressor gene p21 [34–36]. In the present study, we tested RNAa activity of these biotinylated miR-373 in EJ and 5637 cells, linking a biotin to the 5′-end of miR-373 (miR-373-5′-Bio) abolished activation of E-cadherin, whereas biotin labeling at the 3′-end of miR-373 did not affect miR-373’s activating activity. This result is consistent with previous observations [19].

MiRNAs have been reported to play key roles in carcinogenesis and manipulating signal pathways in BCa [37]. Study verified that complementary sequence of specific sits is one of the prerequisites of miRNAs suppression or activation [37, 38]. However, even fully complementarity may not always represent bona fide miRNAs target region without experimental validation [39]. In previous study, we screened out 4 miRNAs which highly complementary to p21 promoter sequences [40]. However, there are only 3 miRNAs were validated to activate p21 expression via targeting the promoter, thereby inhibiting the proliferation and metastasis of human BCa cells [35, 41]. The miRNA with a higher complementary score failed to activate p21 expression, probably because different target sites had different thermodynamic properties, distance from TSS and chromatin/DNA accessibility [42]. Li LC have found that the intrinsic conditions of targeted gene promoters (e.g., DNA methylation) may impair RNAa regulation [12]. The differences in promoter environments of different cell types may affect the number of target genes. The examples of differential induction of target genes were detected in HCT-116, LNCaP and PC-3 cells, in which CSDC2 and E-cadherin were differentially susceptible to gene induction by miR-373 [13].

However, it is unfortunately that the precise mechanism of RNAa remains largely unclear [33, 43]. So far, sifting action ncRNAs target sites from target gene promoter is still a hit-or-miss process [42]. Therefore, further research is required to improve the target forecasting and convenient to gain preferable RNAa. In the current study, we mainly focus on exploring whether miR-373 possessed the ability to activate E-cadherin expression and then suppress cells proliferation, migration and invasion in human BCa cells.

## MATERIALS AND METHODS

### Tissue samples

Human BCa tissues and adjacent noncancerous bladder mucosal tissues were obtained from 40 patients undergoing radical cystectomy. The samples were collected between Jan. 2014 and Jan. 2016 at Tongji Hospital, Tongji Medical College, Huazhong University of Science and Technology (Wuhan, China) after informed consent and Ethics Committee’s approval. And all relevant methods were performed according to the relevant guidelines and regulations. The diagnoses were based on pathology reports. Tissue samples were cryopreserved in liquid nitrogen.

### MiRNA and recombinant lentivirus

The miRNA mimics and 5′- or 3′- biotin linked miRNA mimics were synthesized by RiboBio Co., Ltd. (Guangzhou, China). A short interfering RNA (siCDH1) was used to knockout E-cadherin expression and a dsControl which lacks of significant homology to all known human sequences was synthesized and used as a negative control [12, 44]. Besides, Lenti-miR-373 and Lenti-dsControl were structured by GenePharma (Shanghai, China). The sequences of the custom dsRNAs and miRNA are listed in Supplementary Table 1.

### Cell culture, transfection with miRNA and dsRNA and infection with lentivirus

The human BCa cell lines 5637, EJ and T24 (ATCC, Manassas, VA, USA) were cultured in RPMI 1640 medium (Hyclone, USA) supplemented with 10% fetal bovine serum (FBS) (Gibco, Rockville, MD, USA) in a humidified atmosphere with 5% CO2 at 37°C. The day before transfection BCa cells were seeded on six-well plates, both dsRNA and miRNA were transfected at a final concentration of 50nM with Lipofectamine RNAiMax (Invitrogen, USA) according to the manufacturer’s instructions.

Lenti-miR-373 was used to enforce miR-373 expression following infection into EJ cells according to the manufacturer’s protocols. Lenti-dsControl served as a negative control. Fluorescence intensity was observed at 72-96 h after infection. Then cells were digested and reseeded into new plates for further experiments.

### ChIP assay

The ChIP assay was conducted using a ChIP assay kit (Millipore, USA) and was consistent with the manufacturer’s instructions. 5637 and EJ cells were transfected with biotin-labeled miRNA or dsRNA 72 hours later. About 3×10^6^ cells were used for one separate immunoprecipitation. The RNase inhibitor (Thermo, USA) was used to avoid degradation of RNAs with a final concentration of 50 units/ml. 1% formaldehyde (Sigma, USA) was used to cross-link chromatins for 10 minutes at 37°C. Then the fixed cells were washed and re-suspended in SDS lysis buffer by sonication (30-35 sets of 5 seconds pulses). Chromatins were precleared with protein A agarose/Salmon sperm DNA and immunoprecipitated with well-characterized biotin antibody (Santa Cruz Biotechnology, USA) and IgG (Millipore, USA) overnight at 4°C. Then the antibody/antigen/DNA complex was collected and reversed cross-linked. DNA was column purified (Omega bio-tek, USA) and used as template for qRT-PCR. A list of primers is available in Supplementary Table 2.

### RNA isolation, RT-PCR, quantitative real-time PCR and miRNA analysis

Total RNA was extracted from the tissue and cells with TRIzol reagent (Invitrogen, Carlsbad, CA, USA) according to the manufacturer’s protocol. RNA was reversely transcribed into cDNA using Takara reverse transcription kit (Takara, China) following manufacturer’s instructions. The cDNA was amplified by SYBR Premix Ex Taq II (Takara, China) and conducted on the Mx3000P instrument (Stratagene, USA). All the primers used in this study were offered by Invitrogen (Shanghai, China) and listed in Supplementary Table 2. The levels of gene expression were calculated by relative quantification using GAPDH or U6 small nuclear (snRNA) as the internal reference genes. All reactions were run in triplicate. Besides, the mRNAs’ PCR products were also analyzed on 1.5% agarose gels and visualized.

### Protein extraction and western blotting analysis

Total proteins were extracted using radioimmunoprecipitation assay (RIPA) buffer supplemented with protease inhibitor Cocktail (Roche, Switzerland). Total cell lysates were separated by 10% sodium dodecyl sulfate polyacrylamide gel electrophoresis (SDS-PAGE) and transferred to polyvinylidene fluoride (PVDF) membranes (Roche, Switzerland). Membranes were blocked with 5% bovine serum albumin (BSA) (Sigma-Aldrich, USA) at room temperature for 1h, and then incubated overnight at 4 °C with primary antibodies included Cyclin D1 (1/2000) (Affinity, USA), C-myc (1/1000) (Affinity, USA), MMP2 (1/1000) (Boster, China), E-cadherin (1/1000) (BD Biosciences) and β-actin (1/500) (Boster, China). Then, the membranes were incubated with secondary antibody after three times wash and visualized with enhanced chemiluminescence (ECL) assay kit (Millipore, USA).

### Cell growth assay

Cells were reseeded on 96-well plates after transfected with miRNA mimics. Then, cell viability was assessed by the 3-(4,5-dimethylthiazol-2-yl)-5-(3-carboxymethoxyphenyl)-2- (4-sulfophenyl)-2H-tetrazolium inner salt (MTS) method (Promega, USA) at daily interval for five days upon treatments, according to the manufacturer’s instructions. At each time point, MTS was added and then incubated for 2 h at 37°C. Absorbance was measured at 490 nm.

### Clonogenic survival assay

For colony formation assay, the cells were reseeded at approximate 1000 cells per well in 6-well plates after transfection and cultured 10 days. Then, the colonies were fixed with methanol and stained with 0.5% crystal violet (Sigma, USA) for 30 min at room temperature. The equation: colony formation rate = number of colonies/number of seeded cells × 100% was used to calculate colony formation.

### 5-Ethynyl-2′-deoxyuridine proliferation assay

5637 and EJ cells were reseeded in 96-well plates at 72h post-transfection, a Cell-Light EdU DNA cell kit (Ribobio, China) was used to assess cell proliferation ability according to manufacturer’s protocol. Briefly, the cells were incubated with 50 mM of 5-ethynyl-2′- deoxyuridine (EdU) for 3 hours. After being fixed with 4% paraformaldehyde and treated with 0.5% Triton X-100 for 15 min, cell nuclei were stained with DAPI (Sigma, USA) at a concentration of 1 mg/ml for 30 minutes. The proportion of incorporated EdU-labeled cells was determined by fluorescence microscopy.

### Wound healing assay

Approximate 5 × 10^5^ cells were plated in a 6-well plate after transfection. With incubation for about 12h, the confluent cells monolayers were scratched with a 10 μL pipette tip. Then the cells were cultured in serum-free medium in humidified incubator. The migrated distances were observed and pictured at 0 h, 12 h and 24 h post-scratch, respectively.

### Transwell cell migration and invasion assay

The capability of cell motility was analyzed using a the 24-well Boyden chamber with 8.0-μm pore size polycarbonate membrane insert (Corning, USA). For cell invasion assay, the membrane was pre-coated with matrigel (BD Biosciences, USA) to form a matrix barrier. At 72 h post-transfection, cells were seeded on the upper chamber with 200 μL serum-free medium. 600 μL of medium with 10% FBS was added into lower chamber as a chemoattractant. 24 h later, the membranes were fixed and stained with 0.5% crystal violet (Sigma, USA). The non-motile cells at the top of the membranes were removal with cotton swabs, at least 3 randomly selected visual fields were counted with 200× magnification under a microscope.

### *In vivo* tumorigenicity assay and experimental liver and lung metastasis model

Four-week-old male BALB/c-nude mice were offered by Hua Fukang Biological Technology Company Limited (Beijing, China). All nude mice were cared under specific pathogen free (SPF) condition according to NIH Animal Care and Use Committee guidelines in the Experiment Animal Center of the Tongji medical college of Huazhong University of Science and Technology (Wuhan, China).

EJ cells (about 5 × 10^6^, 200 μL) infected with Lenti-miR-373 or Lenti-dsControl were injected subcutaneously into the right back of male BALB/c-nude mice, respectively. Caliper were used to measure tumor length and width every 3 days for 28 days. Tumor volume was calculated according to the formula: V = length × width^2^ × 0.5. Then, animals were sacrificed 28 days after injection and tumors were weighed.

For *in vivo* metastasis assay, treated EJ cells (2 × 10^5^) were suspended in 100μl of PBS and injected into the tail vein of male BALB/c-nude mice. After 30 days injection, the incidence of metastases was assessed by imaging of mice for bioluminescence using the Living Image software (Xenogen, USA). The photon emission level was used to assess the relative tumor burden in the mice lungs, and the number of liver metastases was estimated tumor metastasis ability.

### Statistical analysis

SPSS 13.0 software (SPSS Inc., Chicago, IL, USA) was used to analyzed the data. All data were presented as the mean ± standard deviation (SD) for three independent experiments. Statistical analysis between groups was performed using unpaired Student’s *t* test and 1-way ANOVA. Pearson correlation coefficient was used to analyzed the correlation between variables. Survival curves were constructed by the Kaplan-Meier method, and log-rank test was used to compare the curves. The Cox regression model was used to simultaneously adjust potential prognostic variables. P < 0.05 was considered to be statistically significant (*P<0.05; (**P<0.01; ***P<0.001).

## CONCLUSIONS

On the whole, our research provides direct evidence that miR-373 has the ability to activate the expression of E-cadherin through targeting specific complementary motifs of the promoter region in human BCa cells. What’s more, miR-373 significantly suppresses the proliferation and metastasis capacity of BCa cells mainly by stimulating E-cadherin expression. Our research fully demonstrates that miR-373 possesses a potential therapeutic application for BCa by targeting activation of E-cadherin. Nevertheless, further study is required to clarify the exact mechanism of RNAa and expand the application domain of miR-373 in different type tumors therapeutics.

## SUPPLEMENTARY MATERIALS TABLES



## Abbreviations

BCa: Bladder cancer
miRNA: MicroRNA
siRNA: Small interfering RNA
EMT: Epithelial-mesenchymal transition
ChIP: Chromatin immunoprecipitation assay
ncRNAs: Noncoding RNAs
RNAa: RNA activation
saRNAs: small active RNAs
TSS: transcription start site
MMP: matrix metalloproteinase
dsRNAs: Double-stranded DNAs
SDS-PAGE: Sodium dodecyl sulfate polyacrylamide gel electrophoresis
PVDF: polyvinylidene fluoride
BSA: Bovine serum albumin
T/N: Tumor/Normal
RT-PCR: Reverse transcription polymerase chain reaction
SD: standard deviation
MTS: 3-(4,5-dimethylthiazol-2-yl)-5-(3-carboxymethoxyphenyl)-2-(4- sulfophenyl)-2H-tetrazolium inner salt
GAPDH: Glyceraldehyde-3-phosphate dehydrogenase, E-cadherin: Epithelial cadherin

## References

[1] Siegel RL, Miller KD, Jemal A (2017). Cancer Statistics, 2017. CA Cancer J Clin.

[2] Torre LA, Bray F, Siegel RL, Ferlay J, Lortet-Tieulent J, Jemal A (2015). Global cancer statistics, 2012. CA Cancer J Clin.

[3] Feng H, Zhang W, Li J, Lu X (2015). Different patterns in the prognostic value of age for bladder cancer-specific survival depending on tumor stages. Am J Cancer Res.

[4] Babjuk M, Oosterlinck W, Sylvester R, Kaasinen E, Böhle A, Palou-Redorta J, Rouprêt M, European Association of Urology (EAU) (2011). EAU guidelines on non-muscle-invasive urothelial carcinoma of the bladder, the 2011 update. Eur Urol.

[5] Stenzl A, Cowan NC, De Santis M, Kuczyk MA, Merseburger AS, Ribal MJ, Sherif A, Witjes JA, Lebret T (2012). Reply to Santhanam Sundar’s letter to the editor Re: Arnulf Stenzl, Nigel C. Cowan, Maria De Santis, et al. Treatment of muscle-invasive and metastatic bladder cancer: update of the EAU guidelines. Eur Urol 2011;59:1009-18. Eur Urol.

[6] Wu CL, Ho JY, Chou SC, Yu DS (2016). MiR-429 reverses epithelial-mesenchymal transition by restoring E-cadherin expression in bladder cancer. Oncotarget.

[7] Berx G, Cleton-Jansen AM, Nollet F, de Leeuw WJ, van de Vijver M, Cornelisse C, van Roy F (1995). E-cadherin is a tumour/invasion suppressor gene mutated in human lobular breast cancers. EMBO J.

[8] Oka H, Shiozaki H, Kobayashi K, Inoue M, Tahara H, Kobayashi T, Takatsuka Y, Matsuyoshi N, Hirano S, Takeichi M, Mori T (1993). Expression of E-cadherin cell adhesion molecules in human breast cancer tissues and its relationship to metastasis. Cancer Res.

[9] McConkey DJ, Choi W, Marquis L, Martin F, Williams MB, Shah J, Svatek R, Das A, Adam L, Kamat A, Siefker-Radtke A, Dinney C (2009). Role of epithelial-to-mesenchymal transition (EMT) in drug sensitivity and metastasis in bladder cancer. Cancer Metastasis Rev.

[10] Breyer J, Gierth M, Shalekenov S, Aziz A, Schäfer J, Burger M, Denzinger S, Hofstädter F, Giedl C, Otto W (2016). Epithelial-mesenchymal transformation markers E-cadherin and survivin predict progression of stage pTa urothelial bladder carcinoma. World J Urol.

[11] Carrington JC, Ambros V (2003). Role of microRNAs in plant and animal development. Science.

[12] Li LC, Okino ST, Zhao H, Pookot D, Place RF, Urakami S, Enokida H, Dahiya R (2006). Small dsRNAs induce transcriptional activation in human cells. Proc Natl Acad Sci USA.

[13] Place RF, Li LC, Pookot D, Noonan EJ, Dahiya R (2008). MicroRNA-373 induces expression of genes with complementary promoter sequences. Proc Natl Acad Sci USA.

[14] Kang MR, Yang G, Place RF, Charisse K, Epstein-Barash H, Manoharan M, Li LC (2012). Intravesical delivery of small activating RNA formulated into lipid nanoparticles inhibits orthotopic bladder tumor growth. Cancer Res.

[15] Kosaka M, Kang MR, Yang G, Li LC (2012). Targeted p21WAF1/CIP1 activation by RNAa inhibits hepatocellular carcinoma cells. Nucleic Acid Ther.

[16] Wei J, Zhao J, Long M, Han Y, Wang X, Lin F, Ren J, He T, Zhang H (2010). p21WAF1/CIP1 gene transcriptional activation exerts cell growth inhibition and enhances chemosensitivity to cisplatin in lung carcinoma cell. BMC Cancer.

[17] Junxia W, Ping G, Yuan H, Lijun Z, Jihong R, Fang L, Min L, Xi W, Ting H, Ke D, Huizhong Z (2010). Double strand RNA-guided endogeneous E-cadherin up-regulation induces the apoptosis and inhibits proliferation of breast carcinoma cells *in vitro* and *in vivo*. Cancer Sci.

[18] Zhang Y, Zhao FJ, Chen LL, Wang LQ, Nephew KP, Wu YL, Zhang S (2014). MiR-373 targeting of the Rab22a oncogene suppresses tumor invasion and metastasis in ovarian cancer. Oncotarget.

[19] Tanaka T, Arai M, Wu S, Kanda T, Miyauchi H, Imazeki F, Matsubara H, Yokosuka O (2011). Epigenetic silencing of microRNA-373 plays an important role in regulating cell proliferation in colon cancer. Oncol Rep.

[20] Seol HS, Akiyama Y, Shimada S, Lee HJ, Kim TI, Chun SM, Singh SR, Jang SJ (2014). Epigenetic silencing of microRNA-373 to epithelial-mesenchymal transition in non-small cell lung cancer through IRAK2 and LAMP1 axes. Cancer Lett.

[21] Nakata K, Ohuchida K, Mizumoto K, Aishima S, Oda Y, Nagai E, Tanaka M (2014). Micro RNA-373 is down-regulated in pancreatic cancer and inhibits cancer cell invasion. Ann Surg Oncol.

[22] Huang Q, Gumireddy K, Schrier M, le Sage C, Nagel R, Nair S, Egan DA, Li A, Huang G, Klein-Szanto AJ, Gimotty PA, Katsaros D, Coukos G (2008). The microRNAs miR-373 and miR-520c promote tumour invasion and metastasis. Nat Cell Biol.

[23] Huang V, Place RF, Portnoy V, Wang J, Qi Z, Jia Z, Yu A, Shuman M, Yu J, Li LC (2012). Upregulation of Cyclin B1 by miRNA and its implications in cancer. Nucleic Acids Res.

[24] Hu J, Chen Z, Xia D, Wu J, Xu H, Ye ZQ (2012). Promoter-associated small double-stranded RNA interacts with heterogeneous nuclear ribonucleoprotein A2/B1 to induce transcriptional activation. Biochem J.

[25] Janowski BA, Younger ST, Hardy DB, Ram R, Huffman KE, Corey DR (2007). Activating gene expression in mammalian cells with promoter-targeted duplex RNAs. Nat Chem Biol.

[26] Chen Y, Luo J, Tian R, Sun H, Zou S (2011). miR-373 negatively regulates methyl-CpG-binding domain protein 2 (MBD2) in hilar cholangiocarcinoma. Dig Dis Sci.

[27] Yan GR, Xu SH, Tan ZL, Liu L, He QY (2011). Global identification of miR-373-regulated genes in breast cancer by quantitative proteomics. Proteomics.

[28] Liu P, Wilson MJ (2012). miR-520c and miR-373 upregulate MMP9 expression by targeting mTOR and SIRT1, and activate the Ras/Raf/MEK/Erk signaling pathway and NF-κB factor in human fibrosarcoma cells. J Cell Physiol.

[29] Lamouille S, Xu J, Derynck R (2014). Molecular mechanisms of epithelial-mesenchymal transition. Nat Rev Mol Cell Biol.

[30] Lipponen PK, Eskelinen MJ (1995). Reduced expression of E-cadherin is related to invasive disease and frequent recurrence in bladder cancer. J Cancer Res Clin Oncol.

[31] Byrne RR, Shariat SF, Brown R, Kattan MW, Morton RA JR, Wheeler TM, Lerner SP (2001). E-cadherin immunostaining of bladder transitional cell carcinoma, carcinoma in situ and lymph node metastases with long-term followup. J Urol.

[32] Bringuier PP, Umbas R, Schaafsma HE, Karthaus HF, Debruyne FM, Schalken JA (1993). Decreased E-cadherin immunoreactivity correlates with poor survival in patients with bladder tumors. Cancer Res.

[33] Jiao AL, Slack FJ (2014). RNA-mediated gene activation. Epigenetics.

[34] Wang C, Ge Q, Chen Z, Hu J, Li F, Ye Z (2016). Promoter-associated endogenous and exogenous small RNAs suppress human bladder cancer cell metastasis by activating p21 (CIP1/WAF1) expression. Tumour Biol.

[35] Wang C, Chen Z, Ge Q, Hu J, Li F, Hu J, Xu H, Ye Z, Li LC (2014). Up-regulation of p21(WAF1/CIP1) by miRNAs and its implications in bladder cancer cells. FEBS Lett.

[36] Ge Q, Wang C, Chen Z, Li F, Hu J, Ye Z (2017). The suppressive effects of miR-1180-5p on the proliferation and tumorigenicity of bladder cancer cells. Histol Histopathol.

[37] Fendler A, Stephan C, Yousef GM, Jung K (2011). MicroRNAs as regulators of signal transduction in urological tumors. Clin Chem.

[38] Jia AY, Castillo-Martin M, Bonal DM, Sánchez-Carbayo M, Silva JM, Cordon-Cardo C (2014). MicroRNA-126 inhibits invasion in bladder cancer via regulation of ADAM9. Br J Cancer.

[39] Saito T, Saetrom P (2010). MicroRNAs—targeting and target prediction. N Biotechnol.

[40] Betel D, Wilson M, Gabow A, Marks DS, Sander C (2008). The microRNA.org resource: targets and expression. Nucleic Acids Res.

[41] Chen Z, Place RF, Jia ZJ, Pookot D, Dahiya R, Li LC (2008). Antitumor effect of dsRNA-induced p21(WAF1/CIP1) gene activation in human bladder cancer cells. Mol Cancer Ther.

[42] Portnoy V, Huang V, Place RF, Li LC (2011). Small RNA and transcriptional upregulation. Wiley Interdiscip Rev RNA.

[43] Zheng L, Wang L, Gan J, Zhang H (2014). RNA activation: promise as a new weapon against cancer. Cancer Lett.

[44] Srsen V, Gnadt N, Dammermann A, Merdes A (2006). Inhibition of centrosome protein assembly leads to p53-dependent exit from the cell cycle. J Cell Biol.

