# Supplementary material for: Enhancing E-cadherin expression via promoter-targeted miR-373 suppresses bladder cancer cells growth and metastasis
Source: Oncotarget. 2017 Sep 30;8(55):93969–83. doi: 10.18632/oncotarget.21400 (PMC5706848; doi:10.18632/oncotarget.21400)
Supplement: Supplementary file 1 [file oncotarget-08-93969-s001.pdf]

Enhancing E-cadherin expression via promoter-targeted miR-373 suppresses bladder cancer cells growth and metastasis

SUPPLEMENTARY MATERIALS

Supplementary Table 1: Sequences for dsRNA and siRNA used in present study

Synthesized RNAs	RNA sequences
miR-373 (S)	ACUCAAAAUGGGGGCGCUUUCC
miR-373 (AS)	GAAGUGCUUCGAUUUUGGGGUGU
dsControl (S)	ACUACUGAGUGACAGUAGA[dT][dT]
dsControl (AS)	UCUACUGUCACUCAGUAGU[dT][dT]
siCDH1 (S)	GGCCTGAAGTGACTCGTAA
siCDH1 (AS)	CCGGACUUCACUGAGCAUU

Supplementary Table 2: Primers used in this study

Name	Sequences (5′-3′)	Assay used for
E-cadherin (S)	ACCAGAATAAAGACCAAGTGACCA	PCR
E-cadherin (AS)	AGCAAGAGCAGCAGAATCAGAAT	PCR
GAPDH (S)	TCCCATCACCATCTTCCA	PCR
GAPDH (AS)	CATCACGCCACAGTTTCC	PCR
Cyclin D1 (S)	GCTGCGAAGTGGAAACCATC	PCR
Cyclin D1 (AS)	CCTCCTTCTGCACACATTTGAA	PCR
C-myc (S)	CTGCTTAGACGCTGGATTT	PCR
C-myc (AS)	TCGTAGTCGAGGTCATAGTTC	PCR
MMP2(S)	ATAACCCACCTAGACCCTAAA	PCR
MMP2(AS)	GGAGTCCGTCCTTACCGTCA	PCR
E-cad-728/-623(S)	ATAACCCACCTAGACCCTAAA	CHIP
E-cad-728/-623(AS)	CTCACAGGTGCTTTGCATC	CHIP
E-cad-1235/-1013(S)	GGTCCTCCAGAAATTCCCCAG	CHIP
E-cad-1235/-1013(AS)	GAGAAGGTCCTTGGAGACA	CHIP
GAPDH (S)	TACTAGCGGTTTTACGGGCGCACGT	CHIP
GAPDH (AS)	TCGAACAGGAGGAGCAGAGAGCGAA	CHIP
dsControl(S)	CCCGGGGTCTACTGTCTC	PCR
dsControl(AS)	CAGTGCAGGGTCCGAGGTAT	PCR
dsControl(RT)	GTCGTATCCAGTGGGGTCCGAGGT ATTCGCACTGGATACGACACTACT	PCR
miR-373(S)	GGGGAAGTGCTTCGATTTTG	PCR
miR-373(AS)	CAGTGCAGGGTCCGAGGTAT	PCR
miR-373(RT)	GTCGTATCCAGTGCAGGGTCCGAGGT	PCR
	ATTCGCACTGGATACGACACACCC	
U6(F)	CTCGCTTCGGCAGCACATA	PCR
U6(R)	CGAATTTGCGTGTCATCCT	PCR
U6(RT)	CGAATTTGCGTGTCATCCT	PCR
